# Mental rotation with abstract and embodied objects as stimuli: evidence from event-related potential (ERP)

**DOI:** 10.1007/s00221-020-05734-w

**Published:** 2020-01-22

**Authors:** Petra Jansen, Anna Render, Clara Scheer, Markus Siebertz

**Affiliations:** Faculty of Human Science, University of Regensburg, Universitätsstraße 31, 93053 Regensburg, Germany

**Keywords:** Chronometric mental rotation task, Sex differences, Embodiment, EEG

## Abstract

This study investigated sex differences in performance and neuronal activity in a mental rotation task with abstract and embodied figures. Fifty-eight participants (26 females and 32 males) completed a chronometric mental rotation task with cube figures, human figures, and body postures. The results are straightforward: depending on angular disparity, participants had a faster reaction time and a higher accuracy rate for embodied stimuli compared to cube figures. The electroencephalogram (EEG) activity pattern showed a higher negative amplitude modulation in the frontal electrodes for females compared to males during the late (400–600 ms) time interval. From 200 to 400 ms after stimulus onset, there was a different activation pattern in the parietal and central electrodes, whereas frontal electrodes did not show differences between embodied and abstract stimuli. From 400 to 600 ms after stimulus onset, there was a different pattern in the central and frontal electrodes but not in the parietal areas for embodied figures in compared to cube figures. Concluding, even though there were no sex differences in the behavioral data, the EEG data did show alterations at the late time interval. Thus, the disparate results regarding sex differences that depend on the type of analysis (behavioral versus neurophysiological) should be more thoroughly investigated. Furthermore, the difference in processing embodied stimuli in an object-based mental rotation task could be confirmed in EEG activity pattern for the first time.

## Introduction

Spatial abilities are highly relevant for everyday activities such as navigation and are also related to, for example, mathematical ability (Xie et al. 2019). According to Uttal et al. (2013), spatial abilities can be differentiated according to two dimensions: extrinsic versus intrinsic and static versus dynamic. One of the most investigated spatial abilities is the intrinsic dynamic ability of mental rotation, in which an object is rotated in one’s own mind (Shepard and Metzler 1971). Mental rotation differs from the spatial ability of perspective taking, which can be classified as extrinsic and dynamic. Two different types of mental rotation transformations are often described: object-based (allocentric, Klatzky 1998) and egocentric mental transformations (Zacks et al. 2000).

### Object-based versus egocentric mental rotation transformations

In the object-based mental rotation transformation task, two stimuli (in normal or mirror-reversed form) are presented. The participants have to decide whether an object is a mirrored reversed version of the other object or not. In the egocentric transformation, e.g., human figures raising their arm (or pictures of hands) are presented and the participants must decide whether it is the left or right arm (or the left or right hand). Pictures of human bodies or parts of human bodies as stimuli (e.g., pictures of hands) in a mental rotation task are called embodied, because the knowledge of the body and its sensorimotor consequences is used for object recognition and transformation (Amorim et al. 2006). It has long been claimed that pictures of abstract or non-human objects, such as cube figures, are processed with an object-based mental transformation, whereas pictures of human bodies or body parts should be processed with an egocentric perspective-based mental transformation (Zacks and Tversky 2005). These two types of mental rotation transformations are linked to different cognitive processes. In object-based transformations, the observer’s position remains fixed, and the object is moved mentally in relation to the surrounding environment, whereas in egocentric mental rotation transformations, one’s own perspective is changed mentally to imagine rotating one’s own body in relation to the stimuli (Voyer et al. 2017). However, egocentric and object-based mental rotation tasks confound the stimulus type (embodied versus non-embodied) and the task instruction (left–right judgment vs. same-different judgment). Most of the egocentric mental rotation studies use embodied stimuli, while most of the object-based studies use abstract objects. Voyer et al. (2017) demonstrated that the influence of the transformation type involved in mental rotation could be examined with the same set of stimuli simply by modifying task instructions. This endeavour can be accomplished by applying a left–right (egocentric) or a mirrored-non-mirrored (object-based) task decision thus making stimulus differentiation unnecessary. In the next section, we will first present the behavioral and neuropsychological results in object-based transformations with mostly abstract stimuli. Subsequently, we will present the results with egocentric transformations and embodied stimuli.

### Neuropsychological effects in object-based mental rotation transformations with abstract objects

The debate on the localization of the mental rotation process in object-based tasks indicates that differential activation for this task is mostly observed within parietal cortical areas (Jordan et al. 2001). A variety of studies investigated neuronal activity during a mental rotation task by analyzing event-related potentials [ERPs, e.g., (Heil and Rolke 2002)]. In general, an early phase of visual object recognition (200–500 ms) is followed by a later phase of mental rotation (400–700 ms). For example, Milivojevic et al. (2009) showed an increase in parietal negativity that commences approximately 400 ms after stimulus onset in both hemispheres and continues until 550 ms over the right hemisphere and until 610 ms over the left hemisphere. The authors concluded that the hemispheric asymmetries are reflected by a faster processing of right parietal rather than of the right hemisphere in general. Schendan and Lucia (2009) also confirmed parietal negativity during mental rotation. Furthermore, their results demonstrated that visual object cognition processes proceed from 200 to 500 ms but also overlap with the initial phase of mental rotation from 500 to 700 ms. The frontocentral N350, which indexes visual object cognition processes, appears more negatively at frontopolar sites between 200 and 700 ms. This result is consistent with psychological models of mental rotation, assuming an overlap between the late phase of object cognition and the early phase of mental rotation.

### Neuropsychological effects in egocentric mental rotation transformations with embodied objects

The results of electroencephalogram (EEG/ERP) egocentric mental rotation transformations tasks are not easy to compare, because they often use different experimental paradigms. However, the results of those studies can be summarized according to an early (around 200–400 ms after stimulus onset) and late (around 400–800 ms after stimulus onset) cognitive processing state. Effects in the early cognitive processing state were found in the following studies: Schwabe et al. (2009) reported a bilateral temporoparietal and frontal activation at approximately 330–420 ms after stimulus onset; the time was dependent on rotation angle in an egocentric task (participants have to decide whether a virtual rotated figure raises the left or right arm). Arzy et al. (2006) examined the EEG activity in an egocentric task (called own body transformation task by the authors) and a mirror task with schematic front- or back-facing human figures. In this mirror task, participants have to imagine themselves at their habitual intracorporeal position or embodied self-location. Therefore, schematic human figures were shown on a monitor, but in contrast to other mental rotation tasks, participants were instructed to imagine that the schematic figure (as shown on the computer screen) was their mirror reflection, as seen from their habitual point of view. Inverse solutions were applied in their study and their data indicated activation (318 ms) in the left extrastriate body area (EBA) for the MIR task and a later activation for the right temporoparietal junction (TPJ) and left EBA at 367 ms. In another study, the mental rotation of body parts is observable at 310–380 ms, with strong activation of left parietal regions (Overney et al. 2005). In additional study with hands as the stimuli, ERPs showed activation in the right parietal (388 ms), bilateral parietal (556 ms), and left frontal area (900 ms) (Thayer et al. 2001).

### Sex differences in mental rotation tasks

A major topic in the field of mental rotation research is the potential existence of sex differences. On a behavioral level, there is an ongoing debate as to whether and how sex influences mental rotation tests (Jansen-Osmann and Heil 2007). Most studies that examine sex differences are conducted with object-based transformations. Hirnstein et al. (2018) assumed that a considerable amount of studies and meta-analyses reveal a male advantage in the behavioral performance in mental rotation tasks with a Cohen’s *d* of 0.56 (Voyer et al. 1995; Zell et al. 2015) and 0.73 (Linn and Petersen 1985).

There are two opposing positions concerning the origin of neuronal differences during mental rotation with respect to sex. Some assume that the ability to mentally rotate objects arises from a general sex-dependent hemispheric asymmetrical processing, as observed in other cognitive tasks (for this general gender-dependent hemispheric asymmetrical processing [see, for example, (Davidson 1995; Hellige 2001; Ocklenburg and Güntürkün 2018)]. Regarding mental rotation, a more bilateral brain activity has been demonstrated for men, whereas women’s brain activity was clearly lateralized to the left (Pellkofer et al. 2014). These data support gender-dependent hemispheric asymmetrical processing. In their review, Hirnstein et al. (2018) discussed that the stronger right-hemispheric asymmetry in males apparently only emerges if specific conditions are met: A flanker stimulus is similarly presented with the mental rotation stimulus (Gootjes et al. 2008), the sample is extended to children (5 years old Hahn et al. 2010), or if adequate stimuli (polygons with five or six vertices) and a large sample size are included (Pellkofer et al. 2014). Other studies found the same activation pattern for both sexes while solving a mental rotation task (Beste et al. 2010; Johnson et al. 2002).

### Goal of this study

The investigation of both kinds of stimuli—embodied and abstract—in an object-based mental rotation task monitored by ERPs and with respect to sex is lacking. Previous studies focused on either one type of stimuli, an egocentric task instruction, or they did not include neurophysiological measurements. Therefore, we would like to close this knowledge gap by investigating potential differences between embodied and abstract stimuli using an object-based task instruction in electrophysiological activation and examine whether any differences are influenced by sex. To exclude the effect of different stimulus features, we used the stimuli from Amorim et al. (2006) who provided cube figures with body characteristics and created two new kinds of stimuli labeled body postures and human figures (Jansen et al. 2012). The participants’ task was to mentally rotate the objects and to decide if the objects were mirror-reversed (called “different”) or non-mirror-reversed (called “same”).

### Hypotheses

- For behavioral data, we predict a replication of a shorter reaction time and higher accuracy with embodied compared to cube figures (see (Amorim et al. 2006; Jansen et al. 2012). According to Voyer and Jansen (2016), if there is a sex difference in a mental rotation task with abstract and embodied stimuli, this effect will be relatively small.
- For electrophysiological activity, it is the first study to examine EEG activity in an object-based mental rotation transformation task using abstract and embodied stimuli. We predict a difference in the activation pattern between embodied and abstract stimuli caused by an easier processing of embodied stimuli. The differences might occur in the parietal cortex (abstract figures are processed in the parietal cortex (Jordan et al. 2001; Milivojevic et al. 2009; Schendan and Lucia 2009), but could also be visible in the frontal area (Schwabe et al. 2009). Regarding sex differences in EEG data with abstract and embodied stimuli, the results regarding lateralization are mixed, and due to the different experimental set-ups in former studies, no directed hypothesis could be formulated (Hirnstein et al. 2018).

## Methods

### Participants

67 participants (33 male, age *M* = 23.48, SD = 3.40; 34 females *M* = 21.06, SD = 4.31) were tested for the experiment. Participants were recruited in university courses of the faculty of human science, pedagogic, and sport science. They were informed about the purpose of the study, gave their written informed consent prior to participation, and received course credit for their participation. The experiment was conducted according to the ethical guidelines of the Helsinki Declaration. Data were analyzed anonymously. Due to artifacts of the EEG, nine participants (seven females, one male) had to be excluded from the analysis. From the remaining 58 participants, 27 were female (age: *M* = 21.04, SD = 4.86) and 31 were male (*M* = 23.56, SD = 3.43). According to Hirnstein et al. (2018a) sample of 48 participants (24 males, 24 females) is sufficient to find a significant sex difference in a mental rotation task with 80% power.

## Materials and procedure

### Behavioral data

**Fig. 1:**
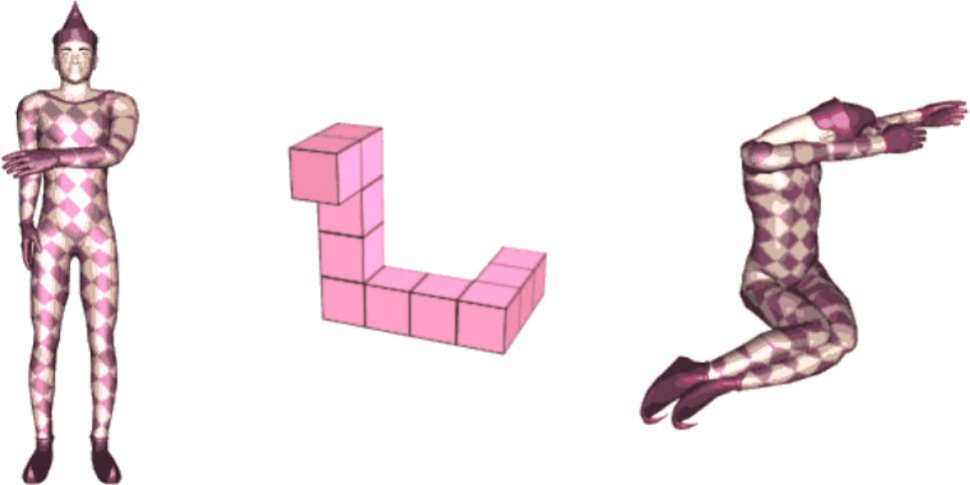
Exemplary stimuli used in the experiment

Mental rotation test. The cMRT was performed on a laptop using Presentation software (version 20.0; Neurobehavioral Systems Inc., Berkeley, California, USA) with a 15.6′ monitor located ∼60 cm in front of the participant, while EEG was monitored continuously. Participants viewed two three-dimensional figures (pairwise), which were centrally presented on the screen, and decided whether the figures were the same or different (mirror-reversed) by clicking the right mouse button for “same” and the left mouse button for “mirrored”. As soon as an answer was given, the next stimuli appeared on the screen. There were three kinds of figures: Cube figures, human figures, and body postures developed by Amorim et al. (2006), see Fig. 1. Each type of stimulus was presented in a separate block (three blocks in total); block orders were randomized across participants. The cMRT consisted of six blocks with 42 trials each (total of 252 randomized trials); half of the trials were identical pairs, the other half mirror-reversed pairs. The pair of stimuli was presented in seven different angular disparities of 0°, 30°, 60°, 90°, 120°, 150°, and 180°. Each figure had a dimension of 400 × 400 pixels. A practice block of 36 trials with feedback preceded the main experiment. During the main experiment, self-controlled pauses were provided after 14 trials. All pairs of stimuli, which were rotated in picture plane (a roll rotation), were colored in pink as originally developed by Amorim et al. (2006) and displayed in front of a white background.

Statistical analysis: Mirror-reversed items were excluded from analyses, because angular disparity is not unambiguously defined for cube figures (e.g., Jolicœur et al. 1985) and cannot be brought into congruence with another (Shepard and Metzler 1971). Whereas a mirrored figure is clearly unique in shape, depending on the orientation of the mirroring plane, the mirror image appears at different rotated positions. This means that two mirror images deviate by twice the angle at which the two mirroring planes are rotated to each other. Thus, no unique 0°-condition exists and no angular disparity for mirrored stimuli. This problem also occurs in 2D cube figures as rotation of mirror lines also produces rotated stimuli (Jost and Jansen 2020). Therefore, all statistical analyses were restricted to the same/identical items only. For reaction times, only correct responses were considered and reaction times deviating by two from the mean reaction time (of all reaction times for one stimulus type in each angle) were replaced according to the studies of Hahn et al. (2010) and Jansen-Osmann and Heil (2007) through the mean value of the stimulus (at that angle). The reaction times of 66 trials (4.69% with 20 cube figures, 26 body postures, and 20 human figures) were replaced. To analyze reaction times and accuracy, a repeated-measured ANOVA with the between factor sex and the within factors stimuli (3) × angle (7) was conducted.

### Electroencephalography data

*Electroencephalography measurement* For collecting the electroencephalography (EEG) data, 32 Ag/AgCl active electrodes were used (Brain Products, Gilching, Germany), positioned in a recording cap (ActiCap) according to the 10–20 system. Furthermore, four bipolar passive eye electrodes sampled the vertical and the horizontal eye movements. Data were recorded using the BrainVision Recorder 1.21 software (Gilching, Germany) at a continuous sampling rate of 500 Hz. The signal was amplified using the BrainVision QuickAmp USB 40-channels (32 EEG channels) and referenced to an average reference. No online filters were applied, and electrical impedance was always maintained below 20 kΩ. Electrical noise was avoided by running all technical advices on battery.

*Statistical analysis* The EEG data were analyzed with the Brain Vision Analyzer 2.1.1 (Brain Products). For this analysis, we use the so-called current source density (CSD) method (Kayser and Tenke 2015; Pascual-Marqui et al. 1988). The sampling rate was reduced to 250 Hz. A band pass filter from 0.1 to 20 Hz, but no notch filter was applied. Dividing the data into fragments around stimulus presentation (− 100 to 2000 ms) generated segments of 2100 ms length. A semiautomatic artifact rejection marked segments as not satisfactory according to set criteria: maximal allowed voltage step of 80 µV/ms; maximal allowed difference between values of 250 µV (interval length 400 ms); minimal/maximal allowed amplitude of ± 175 µV; and activity lower than 0.5 µV for 150 ms. Ocular artifacts were corrected according to Gratton et al. (1983). We evaluated event-related potentials by averaging segments of correct responses to non-mirrored stimuli for each participant. The averaged segments were CSD-transformed making the values reference-free. The mean voltages per area of the electrodes were analyzed in the time interval after stimulus onset between 200–400 ms and 400–600 ms. Given previous result, we selected the 200–400 ms as the “early activation phase”. Additionally, the later interval was analyzed, because, for example, Thayer et al. (2001) demonstrated a bilateral activation pattern for pictures of body parts as stimuli at 600 ms after stimuli onset. According to the study of Hahn et al. (2010), the mean voltages of the *F* (*F*3, *F*4), *C* (*C*3, *C*4), and *P* (*P*3, *P*4) electrodes were analyzed. If sphericity was violated, the relevant results were Greenhouse–Geisser corrected. All post hoc tests were Bonferroni corrected and significance level was set to *p* < 0.016 for testing effects concerning the three different stimuli.

To analyze EEG activity, a repeated-measured ANOVA with the between factor sex and the within factors stimuli (3, cube figures, human figures, body postures), lateralization (2, left, right), and electrode (3, electrodes: *F*, *C*, *P*) was conducted. Angle was not examined in the EEG analysis due to lack of specific hypotheses as a factor and to reduce the complexity of the design. All results with stimuli as a factor will be explained in more detail.

## Results

### Behavioral data

**Fig. 2:**
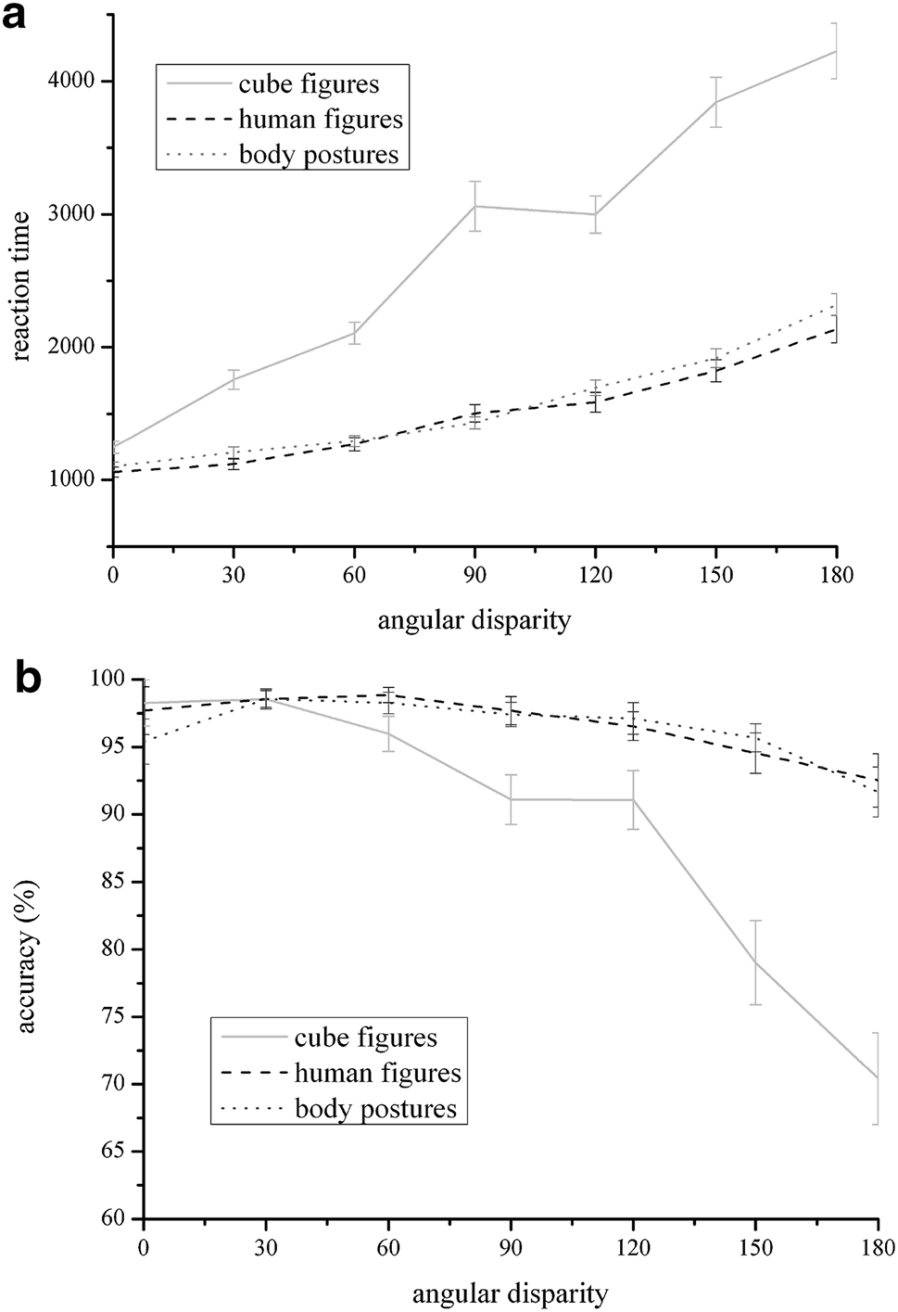
The *x*-axis represents angular disparity between the two mental rotation objects, while the *y*-axis shows the mean of the reaction time (**a**) and the mean of accuracy (**b**). The data show different performance outcomes depending on stimulus type. For all stimuli, an increase in RTs (**a**) and a decrease in accuracy (**b**) with increasing angular disparity were observed. The most drastic increase in reaction time (decrease in accuracy) was observed for cube figures, while the changes for human bodies and body postures were very small

*Reaction times* The univariate analysis of variance showed a main effect of stimuli *F*(1.242, 69.53) = 171.92, *p* < 0.001, partial *η*^2^ = 0.754. Reaction times were slowest for cube figures (*M* = 2748.08, SD = 860.39), followed by body postures (*M* = 1567.48, SD = 338.05) and human figures (*M* = 1499.94, SD = 414.10). There was also a main effect of angular disparity, *F*(2.318, 129.83) = 204.65, *p* < 0.001, partial *η*^2^ = 0.785. A highly significant linear trend could be demonstrated, *F*(1, 56) = 336.72, *p* < 0.001, partial *η*^2^ = 0.857 which was completed by a quadratic trend, *F*(1, 56) = 11.18, *p* = 0.001, partial *η*^2^ = 0.166 and one of the 6th order, *F*(1, 56) = 19.137, *p* < 0.001, partial *η*^2^ = 0.255. Both two main effects are qualified by a significant interaction between them, *F*(4.647, 260.25) = 45.03, *p* < 0.001, partial *η*^2^ = 0.446. Figure 2 indicates that the reaction time for the three different stimuli followed a different trend pattern: For cube figures a linear trend, *F*(1, 57) = 243.53, *p* < 0.001, partial *η*^2^ = 0.810 and one of 6th order, *F*(1, 57) = 20.745, *p* < 0.001, partial *η*^2^ = 0.267 could be detected, for human figures a linear trend, *F*(1, 57) = 157.03, *p* < 0.001, partial *η*^2^ = 0.734, a quadratic trend, *F*(1, 57) = 11.721, *p* = 0.001, partial *η*^2^ = 0.171 and one of 4th order, *F*(1, 57) = 4.81, *p* < 0.05, partial *η*^2^ = 0.078 was visible, and for body postures, the analysis showed a linear *F*(1, 57) = 319.03, *p* < 0.001, partial *η*^2^ = 0.848, and a quadratic trend, *F*(1, 57) = 48.36, *p* < 0.001, partial *η*^2^ = 0.459.

*Accuracy* Analog to reactions times, there was a main effect for stimuli in accuracy *F*(1.428, 85.58) = 27.53 *p* < 0.001 partial *η*^2^ = 0.330. Accuracy was lower for cube figures *M* = 89.2%, SD = 0.107, than body postures *M* = 96.30%, SD = 0.042 and human figures *M* = 96.63%, SD = 0.052. In addition, there was a main effect for angular disparity, *F*(3.104, 173.83) = 25.76 *p* < 0.001 partial *η*^2^ = 0.315. A highly significant linear trend could be demonstrated, *F*(1, 56) = 54.78, *p* < 0.001, partial *η*^2^ = 0.494, which was qualified by a quadratic, *F*(1, 56) = 25,26, *p* < 0.001, partial *η*^2^ = 0.311 and a trend of 5th order, *F*(1, 56) = 4.61, *p* = 0.036, partial *η*^2^ = 0.076. For the interaction effect for stimuli × angular disparity, *F*(6.016, 672) = 15.61 *p* < 0.001 partial *η*^2^ = 0.218, a different trend dependent on the type of stimuli was indicated: for cube figures, all trends without the cubic and the one of fourth order were significant (all *p* < . 05), for human figures only a linear trend was visible, *F*(1, 56) = 8.51, *p* < 0.01, partial *η*^2^ = 0.132, and for body postures, the analysis showed a linear *F*(1, 56) = 5.97, *p* < 0.05, partial *η*^2^ = 0.096, and a quadratic trend, *F*(1, 56) = 11.32, *p* = 0.001, partial *η*^2^ = 0.168. For the analysis of reaction time and accuracy, there was neither a main effect of sex nor an interaction with the other two factors, all *p* > 0.13.

### EEG data

All EEG mean values represent microvolts per square meter.

### Interval 200–400 ms

Two main effects for the factors electrode, *F*(1.204,67.435) = 85.249, *p* < 0.001, partial *η*^2^ = 0.604 and stimuli, *F*(2, 112) = 3.89, *p* < 0.05, partial *η*^2^ = 0.065 and two interaction effects between electrode × laterality, *F*(2, 112) = 15.289, *p* < 0.001, partial *η*^2^ = 0.214, and electrode × stimuli, *F*(3.276, 183.454) = 2.714, *p* < 0.05, partial *η*^2^ = 0.046 can be reported.

**Fig. 3:**
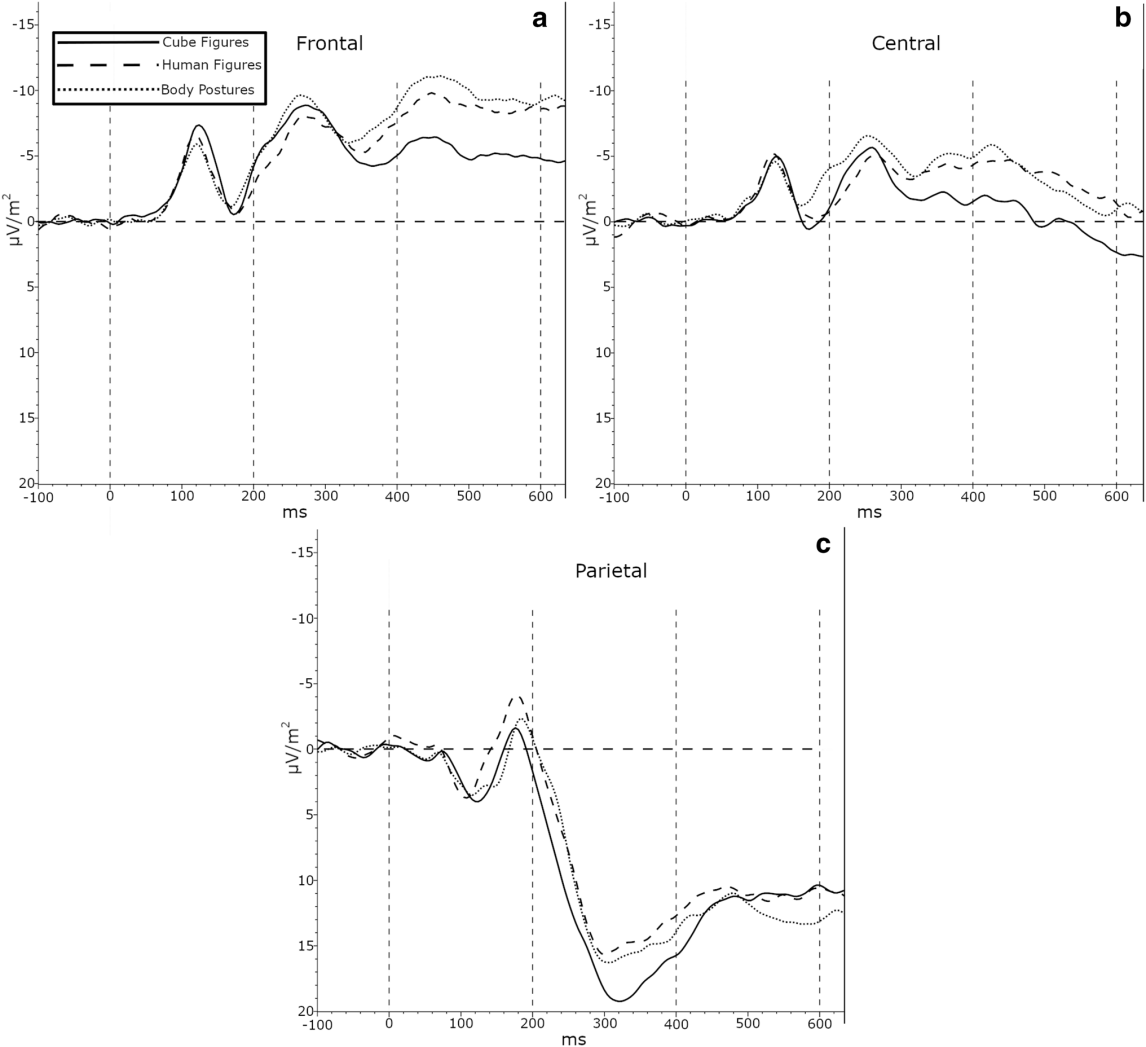
Grand average of the CSD-transformed EEG-signal for segments between 100 ms before to 600 ms after stimulus onset for the three stimulus types pooled over electrodes F3/F4 (**a**), C3/C4 (**b**), and P3/P4 (**c**). The time intervals of interest are marked by vertical dashed lines. For the interval between 200 and 400 ms, body postures lead to a more negative signal than cube figures at the central electrodes, while cube figures lead to a more positive signal than body postures and human figures at the parietal electrodes. During the interval between 400 and 600 ms, body postures and human figures lead to a more negative signal than cube figures at both, the central and the frontal electrodes

First of all, the results showed the most positive amplitude for the cube figures (*M* = 1.51, SD = 5.19), compared to the human figures (*M* = 0.54, SD = 6.82), *t*(56) = 2.14, *p* < 0.05 and body postures (*M* = − 0.95, SD = 5.44), *t*(56) = − 2.721, *p* < 0.01. However, after Bonferroni correction, only the difference between cube figures and body postures was significant. Second, the interaction of stimuli and electrode demonstrated that there was no significant difference between the stimuli at the frontal electrode, *F*(1.184, 103.37) = 0.163, *p* = 0.850, partial *η*^2^ = 0.003, but at the central, *F*(2, 114) = 3.445, *p* < 0.035, partial *η*^2^ = 0.057 and parietal electrode, *F*(2, 114) = 6.568, *p* < 0.001, partial *η*^2^ = 0.103. For the central electrode, only body postures (*M* = − 4.95, SD = 8.18) showed higher negative amplitude than cube figures, (*M* = − 2.89, SD = 7.43), *t*(57) = − 2.719, *p* < 0.01. For the parietal electrode, human figures (*M* = 11.09, SD = 12.04) and body postures (*M* = 11.53, SD = 11.10) showed lower positive amplitude than cube figures (*M* = 14.20, SD = 12.39), *t*(57) = 3.536, *p* < 0.01 for human figures and *t*(57) = − 2.746, *p* < 0.01 for body postures, see Fig. 3.

### Interval 400–600 ms

Two main effects for the factors electrode, *F*(1.283,71.828) = 92.263, *p* < 0.001, partial *η*^2^ = 0.622 and stimuli, *F*(2, 112) = 5.95, *p* < 0.01, partial *η*^2^ = 0.096 and three interaction effects between electrode × sex, *F*(1.283,71.828) = 4.210, *p* < .05, partial *η*^2^ = .070, electrode + laterality, *F*(2, 112) = 7.950, *p* < .001, partial *η*^2^ = .124, and electrode × stimuli, *F*(3.025, 169.399) = 4, 711, *p* < 0.01, partial *η*^2^ = 0.078 can be seen.

First, the results showed the most positive amplitude for the cube figures (*M* = 2.06, SD = 5.84), compared to the human figures (*M* = 0.70, SD = 7.09), *t*(57) = 2.43, *p* = 0.018 and body postures (*M* = 0.786, SD = 5.84). *t*(57) = − 1.99, *p* = 0.05. However, after Bonferroni correction, these differences were not significant. Second, the interaction of stimuli and electrode demonstrated that there was no significant difference between the stimuli on the parietal electrode, *F*(2, 114) = 0.570, *p* = 0.567, partial *η*^2^ = 0.010, but on the central, *F*(2, 114) = 7.609, *p* = 0.001, partial *η*^2^ = 0.118 and frontal electrode, *F*(2, 114) = 10.74, *p* < 0.001, partial *η*^2^ = 0.159. For the central electrode, human figures (*M* = − 3.50, SD = 10.50) and body postures (*M* = − 3.15, SD = 8.33) showed higher negative amplitude than cube figures (*M* = − 0.2667, SD = 8.45), *t*(57) = 3.426, *p* < 0.01 and *t*(57) = 3.187, *p* < 0.01. For the frontal electrode, human figures (*M* = − 8.83, SD = 10.70) and body postures (*M* = − 9.88, SD = 9.39) showed higher negative amplitude than cube figures, (*M* = − 5.43, SD = 7.29), *t*(57) = 3.396, *p* = 0.001 for human figures and *t*(57) = − 4.591, *p* < 0.001 for body postures., see Fig. 3.

Third, the interaction between electrode and sex could be explained by the effect of sex on the frontal electrodes *F*(1, 56) = 4.85, *p* < 0.05, partial *η*^2^ = 0.08, but not on the central, *p* = 0.884 and parietal electrodes, *p* = 0.117. Females (*M* = − 10.47, SD = 9.60) showed higher negative amplitude than males (*M* = − 5.93, SD = 5.87) on the frontal electrodes.

## Discussion

Regarding the behavioral analysis, our study replicated previous results that showed mental rotation tasks with human figures in an object-based mental transformation task which are easier to solve. Participants had a faster reaction time and showed a higher accuracy rate while solving the task with human figures. Furthermore, it has been shown for the first time that abstract and embodied stimuli evoked a different activation pattern in an object-based mental rotation task with a same-different decision in the EEG. Sex differences could not be identified in the behavioral, but were confirmed in the EEG data.

### Mental rotation in an object-based transformation task with abstract and embodied figures

The better behavioral performance in the object-based mental rotation task with embodied compared to abstract figures is consistent with former studies using the same kind of stimuli (Amorim et al. 2006; Jansen et al. 2012; Voyer and Jansen 2016). It supports the embodiment approach. Stimuli that evoke a motor resonance process are easier to process than abstract stimuli (e.g., (Liuzza et al. 2012), which has been shown especially in a mental rotation task (Amorim et al. 2006).

An innovative point highlighted by this study is the use of whole human body stimuli that are put in the same positions as the abstract cubes. This design allows direct comparison of these stimuli. Although other studies provide evidence for differences in mental rotation performance in different stimuli (Dalecki et al. 2012), the fairly new result from this study is that the stimulus types are designed in a similar way; their features are comparable. They only differ in the extent of abstractness and embodiment, causing the differences in performance and neural activation. This difference depends on the time interval chosen. In the earlier time interval, the activity pattern differs between the embodied figures compared to the cube figures at the parietal and central electrodes but not at the frontal ones. In the later time interval, different stimuli did not lead to differential activation in parietal areas, whereas embodied stimuli did lead to more negative amplitude than cube figures in the central and frontal areas. This result adds to the study of Schwabe et al. (2009), which demonstrated frontal activation in a task with human bodies. However, Schwabe et al. (2009) did not compare their results with an object-based transformation mental rotation task (e.g., cube figures). There was no interaction with lateralization; consequently, our results are in accordance with Thayer et al. (2001). These authors revealed that mental rotation with hands as stimuli lead to bilateral parietal activation (among other regions). Their participants had to decide whether the presented rotated hand is right or left, which is an egocentric mental rotation (in their words an OBT, object-based transformation task). In general, the results of this study add to a meta-analysis of brain imaging studies that showed a different activation in the brain network dependent on the kind of stimuli with body versus non-body parts (Tomasino and Gremese 2015). Our study also indicates that the extent of embodiment (body postures versus human figures) in the stimulus does not influence the results in an object-based transformation task. Furthermore, this study indicates that different processing, dependent on the embodiment of stimuli in an object-based transformation, relates to the relevant time interval. In the earlier phase of processing the stimuli, differences appear in the parietal and central area, whereas in the later phase of processing the stimuli, differences can be seen in the central and frontal area. This is in line with a study using hands as stimuli; the ERP activation has been shown earlier in the parietal (388–556 ms) and later in the left frontal area (900 ms) (Thayer et al. 2001).

Another argument supporting differential processing of stimuli in a mental rotation task is the fact that the fixation patterns have been shown by Nazareth et al. (2019) to differ. In their recently published study, the participants’ eye movements during an object-based mental rotation task with abstract figures were tracked (Nazareth et al. 2019). The results (latent profile analysis that combined different eye movement parameters) indicated two distinct eye-patterns: fixating (indicating a holistic strategy) and switching (indicating a piecemeal strategy). The switching eye-pattern was related to high mental rotation performance. Furthermore, the flexibility of the use of a strategy has been calculated (Nazareth et al. 2019). One possibility to detect differences in strategy use would be to investigate if abstract and embodied figures evoke a different eye-pattern strategy or more flexibility in strategy use. Furthermore, eye tracking allows the measurement of the cognitive effort via pupil dilation (Palinko et al. 2010). The task-evoked changes of the pupil diameter can be used as a “psychophysiological index or correlate of cognitive activity” (Campbell et al. 2018). Campbell et al. (2018) used this method to detect sex differences in cube versus hand stimuli finding cube figures to be less cognitively taxing for males, who showed significantly lower pupil dilation. Sex differences could not be identified for the hands stimuli. This approach might also be useful to detect cognitive strain in relation to the degree of embodiment of stimuli.

### Sex differences in an object-based mental rotation task with abstract and embodied stimuli

In general, a main sex effect, as found by Voyer and Jansen (2016), could not be confirmed in the behavioral data. One reason for this discrepancy may be the variation in the use of the human stimuli between the two studies. Voyer and Jansen (2016) presented head cubes (cube with the addition of a head) while we investigated human postures. Nevertheless, the lack of sex differences in this study are consistent with a study from Jansen-Osmann and Heil (2007), who investigated sex differences in mental rotation tasks with five different stimulus types and a sample size of *N* = 360, 72 (36 men and 36 women) for each stimulus condition. They only found sex differences in one (polygons) out of five different stimulus types [letters, cube figures, stimuli from the primary mental ability test of Thurstone (1958), and animal pictures] in the mental rotation task. As in the study of Jansen et al. (2012), the different stimuli in this investigation were presented in separate blocks. This design differs from the randomized presentation of the discrete stimuli in each block in the study of Voyer and Jansen (2016). Thus, we conclude that the issue of possible sex differences in chronometric mental rotation tasks remains unresolved. Whereas sex differences that favor men appear to be stable in psychometric (i.e., paper–pencil) tests, the difference is not evident in the chronometric approach based on reaction time. Seven of the 15 chronometric studies that were included in the meta-analysis of Voyer et al. (1995) showed no sex difference. However, before discussing potential explanations of possible sex differences, such as the psycho-social and biological-neuronal reasons (e.g., (Newcombe 2002), it is necessary to investigate some methodological issues: How relevant is the stimulus material, the kind of presentation, and the time limit for the existence of possible sex differences? The results of possible sex differences in mental rotation tasks appear to depend on the used method, and thus, these aspects must be investigated in greater detail.

The same argument holds true for the interpretation of the EEG pattern considering potential sex differences. The results showed higher amplitude modulation in the frontal electrodes for females compared to males during the late time interval for all stimuli. A different pattern for males and females was also found in another study (Desrocher et al. 1995). Women show greater positivity modulation in parietal (P3) electrodes in several types of tasks. The authors concluded women might use more analytic strategies, including higher cortical involvement, compared to men (Desrocher et al. 1995). Beste et al. (2010) demonstrated difference in the EEG with regard to performance level: The high-performing group exhibited lower amplitudes compared to the low-performing group. The authors used the neural efficiency hypothesis as a possible explanation for the association between higher performance levels and more efficient brain function. Our result regarding sex differences appeared to be independent of lateralization, a finding that is consistent with previous studies (Howard et al. 1992; Pellkofer et al. 2014), but dependent on the cortical side. However, the behavioral data do not indicate differences between the sexes; men and women performed at the same rate.

### Strength and limitations

The strength of the paper is that it investigated for the first time EEG activity in an object-based mental rotation transformation with a same–different decision task using abstract and embodied stimuli. For this object-based transformation task, the differences in reaction time, accuracy rate, and neurophysiological activity could not be attributed to other stimuli features that may have influenced the results. However, one limitation of the study is that the utilized human bodies are not realistic. One possibility could be the use of pictures of living persons. For example, males show a better performance compared to females with these stimuli (Kaltner et al. 2017).

Furthermore, there are other relevant factors that we know which influence mental rotation, such as motor expertise (Voyer and Jansen 2017). A motor expertise effect is often discussed in the framework of the embodied cognition approach. This approach posits that motor and sensory processes are interlinked and share a common representational system (Lakoff and Johnson 1999). Because of this common representational system, it can be assumed that motor expertise is related to cognitive performance as it has been shown for the motor expertise effect in mental rotation (Voyer et al. 2017). Particularly, there has been evidence for a better mental rotation performance of soccer players compared to non-soccer players using the same kind of stimuli (Jansen et al. 2012) as we did in this study. Based on these previous results, we asked our participants to report whether they played any ball sports. However, no significant differences were found in the behavioral and EEG data when the sports activity was included in an ANCOVA. Next to motor expertise, the level of performance (Beste et al. 2010) must be considered in greater detail in future studies.

## Conclusion

This study replicated the effect that mental rotation tasks with stimuli that are in some way embodied are easier to process. Furthermore, it provides a hint that the activation pattern in the EEG is different for abstract and embodied figures. Specifically, the results showed a more positive amplitude modulation in the parietal electrodes for females compared to males during the late time interval. Embodied stimuli in an object-based transformation task led to a different modulation in the parietal and central areas in the earlier time interval and in the frontal and central (but not parietal) areas compared to cube figures during the later time interval (400–600 ms). Neuronal activity and behavioral measurements showed the same patterns in an object-based transformation: abstract and embodied stimuli in a task, which does not require an own body transformation, are processed differently. Furthermore, the disparate results regarding sex depend on the type of analysis (behavioral versus neurophysiological) and should be investigated in greater depth.
